# High-Accuracy and Efficient Classification of Uranium Slag by Origin and Category via LIBS Integrated with Hybrid Machine Learning

**DOI:** 10.3390/s26082522

**Published:** 2026-04-19

**Authors:** Mengjia Zhang, Hao Li, Luan Deng, Rong Hua, Xinglei Zhang, Debo Wu, Xizhu Wang, Xiangfeng Liu, Zuoye Liu, Xiaoliang Liu

**Affiliations:** 1 Jiangxi Province Key Laboratory of Nuclear Physics and Technology, East China University of Technology, Nanchang 330013, China; 2025110518@ecut.edu.cn (M.Z.);; 2 Jiangxi Key Laboratory for Mass Spectrometry and Instrumentation, East China University of Technology, Nanchang 330013, China; 3 High Institute of Advanced Studies, University of Chinese Academy of Sciences, Hangzhou 310024, China; 4 School of Nuclear Science and Technology, Lanzhou University, Lanzhou 730000, China

**Keywords:** uranium slag, laser-induced breakdown spectroscopy (LIBS), machine learning, feature engineering, classification

## Abstract

Accurate classification of uranium slag origin and category is essential for nuclear environmental monitoring and safety. This study presents a hybrid framework combining laser-induced breakdown spectroscopy (LIBS), four preprocessing methods, and five machine learning algorithms for rapid uranium slag classification. A total of nine sample categories were collected from three mining areas, with categories defined by their U concentration levels within each origin. Standard normal variate (SNV), Savitzky–Golay smoothing (SG), and their combinations (SNV-SG, SG-SNV) were applied to evaluate preprocessing effects. To address ultra-high-dimensional spectral data (49,242 points per spectrum), principal component analysis (PCA) and random forest (RF) were employed for feature engineering, integrated with support vector machine (SVM), linear discriminant analysis (LDA), and K-nearest neighbors (KNN) classifiers. Hyperparameter optimization via five-fold cross-validation and Bayesian optimization enhanced accuracy and efficiency. RF-based hybrid models consistently outperformed PCA-based counterparts. Remarkably, the RF-LDA model with SNV-SG preprocessing achieved 100% classification accuracy across all test sets with a processing time of only 10.46 s, demonstrating exceptional discriminative power and computational efficiency. These findings establish that combining RF feature selection with advanced machine learning offers a robust solution for LIBS-based nuclear material classification, with significant implications for both nuclear safety and resource management.

## 1. Introduction

The efficient characterization and safe management of nuclear waste are critical challenges in environmental science and the sustainable development of nuclear energy, with the proper treatment and disposal of uranium slag being particularly important. As the primary byproduct of uranium mining and hydrometallurgical processes, uranium slag typically contains lots of heavy metals and radioactive nuclides such as uranium, thorium, radium and potassium. Improper management of these components can result in their prolonged presence in the environment, posing potential threats to ecosystems and public health [1]. Currently, traditional analytical methods such as atomic absorption spectroscopy (AAS) and inductively coupled plasma optical emission spectroscopy (ICP-OES/MS) face numerous limitations in the practical application to such complex and radioactive materials, including complicated sample pretreatment, large reagent consumption, potential generation of secondary waste, and radiation exposure risks for the analysts. Therefore, the development of a rapid, non-contact, and highly sensitive method for tracing the origin and screening the category of uranium slag is of significant practical importance. As an emerging atomic emission spectroscopy method, laser-induced breakdown spectroscopy (LIBS) enables the detection of elemental constituents based on characteristic atomic and ionic emission lines, and has shown remarkable potential in the analysis of radioactive materials in recent years. It is important to note, however, that conventional LIBS generally cannot distinguish between isotopes of the same element, as the spectral shifts induced by isotopic mass differences are extremely small and typically beyond the resolving power of standard LIBS systems. Despite this limitation, LIBS offers several unique advantages, including minimal sample pretreatment [2], nearly non-destructive analysis [3], real-time rapid response [4], and the ability to detect multiple elements simultaneously [5]. These features make LIBS particularly suitable for the direct, on-site and remote analysis of radioactive waste such as uranium slag, providing a new technical approach for developing safer and more efficient nuclear waste characterization systems [6].

Despite the significant advantages of LIBS, its spectra are often affected by matrix effects, self-absorption, spectral overlap, and background noise [7], leading to complex nonlinear relationships between spectral signals and sample composition [8].

This challenge is particularly pronounced in the analysis of uranium ores [9], which exhibit a complex and variable composition, making traditional quantitative analysis methods based on fixed calibration curves suffer from limited accuracy and generalizability. To address these bottlenecks, combining machine learning algorithms has proven effective for processing such complex spectral data [10,11,12]. Machine learning models, particularly advanced supervised learning algorithms, do not rely on pre-established physical models. Instead, they automatically extract key features and hidden patterns directly from high-dimensional and complex spectral data through a data-driven approach [13], enabling precise identification and classification of samples [14]. Accordingly, this data-driven paradigm has established LIBS as a cornerstone analytical technique within the nuclear sector, providing a robust alternative for rapid characterization in radiologically hazardous environments [15]. Substantial research has validated the versatility of this approach in the elemental analysis of actinides such as uranium and thorium across heterogeneous matrices, from aqueous solutions [16] to powdered substrates and complex geological ores [17]. These diagnostic capabilities are further reinforced by fundamental investigations into the time-resolved expansion dynamics of uranium-bearing plasmas, which offer a theoretical framework for optimizing spectral sensitivity [18]. Consequently, the field is witnessing a strategic shift toward “intelligent spectroscopy”, where the synergy between LIBS and advanced algorithms facilitates high-fidelity nuclear forensics and the trace analysis of fission products [19,20,21]. Beyond the nuclear domain, numerous studies have demonstrated the successful application of machine learning in LIBS data analysis. He et al. [22] combined femtosecond laser ablation spark-induced breakdown spectroscopy with machine learning to achieve high-precision classification of steel alloys. Their research showed that the random forest (RF) model exhibited optimal performance, with an average accuracy of 0.9337 over 100 independent classifications and a micro-average area under the curve (AUC) value of 0.9996 in five-fold cross-validation, significantly outperforming the support vector machine (SVM) and partial least squares regression (PLSR) models. Senesi’s team [23] employed handheld LIBS combined with supervised learning for the geochemical identification of flint samples, comparing the classification performance of discriminant analysis (DA), SVM, and k-nearest neighbors (KNN) algorithms after principal component analysis (PCA) dimensionality reduction. After eliminating the effects of intra-class variation on principal component 1, linear discriminant analysis (LDA) achieved a classification accuracy of 80.85% for five categories of samples, with several discriminant combinations exceeding 90%, and the classification accuracy between Vieste (Defensola B, DEFB) and Peschici (Tagliacantoni, TGC) mining sites reached 100%. Wang et al. [24] proposed an explosive detection method based on LIBS and semi-supervised learning, which effectively differentiated various explosives and plastic interferents by combining label propagation with KNN classification. With only 3.3% labeled samples, the method achieved 100% true positive identification rates for both known and unknown explosives, with a specificity of 99.17%, demonstrating excellent robustness and classification stability.

However, the construction of high-performance machine learning classifiers not only relies on the selection of algorithms but is also largely constrained by the quality of input data [25]. Generally, data preprocessing and feature engineering are widely employed to enhance data quality. Spectral preprocessing (e.g., background correction, data normalization and removal of noise) is a critical step in LIBS analysis, as it aims to minimize or eliminate spectral interference caused by non-chemical factors such as fluctuations in laser energy, sample inhomogeneity, and instrument drift [26]. Appropriate preprocessing not only significantly enhances the stability of model training and classification accuracy but also prevents the adverse effects of data distortion on the results. Meanwhile, feature engineering plays an equally vital role by transforming raw spectral data into a set of informative, non-redundant features that better represent the underlying sample characteristics. Directly processing these high-dimensional data can easily lead to the so-called “curse of dimensionality”. Particularly under conditions of limited sample size, directly handling high-dimensional data not only significantly increases computational burden and reduces training efficiency but also makes the model prone to overfitting noise and irrelevant variables, leading to a decrease in generalization ability. Therefore, feature engineering has become a crucial step in improving data quality and optimizing model performance. This process aims to select the most discriminative feature subset from the original high-dimensional feature space, effectively compressing the data size while retaining core information [27].

Studies have shown that feature engineering can significantly improve model performance, enhance robustness, and increase interpretability. Zhang et al. [28] applied genetic algorithms (GA), competitive adaptive reweighted sampling (CARS), and successive projections algorithm (SPA) for feature engineering on LIBS spectra, reducing the original 14,741-dimensional data to 7001, 965, and 135 dimensions, respectively. Combined with multiple machine learning algorithms, they achieved 100% accuracy in classifying six samples of Bupleurum from different origins. Zhao et al. [29] systematically compared the performance of four variable selection methods, including least angle regression (LAR), CARS, SPA, and GA, in the non-destructive classification of lily bulbs. They found that the logistic regression (LR) model based on 10 key features selected by LAR achieved 100% classification accuracy in both cross-validation and prediction sets. Banerjee’s team [30] used PCA to reduce the dimensionality of LIBS spectra, effectively compressing high-dimensional data into three principal components (PCs). Combined with maximum normalization preprocessing, they achieved accurate classification of eight samples of yellow cake. The spectral range was reduced from 600 nm to 15 nm, significantly improving analysis efficiency while maintaining ≥ 90% classification accuracy. Wang et al. [31] combined LIBS with a variable importance-based random forest (VIM-RF) model for the acidity analysis of iron ore, showing that the VIM-RF model outperformed partial least squares (PLS) and least squares support vector machine (LS-SVM) models. The model’s root mean square error of validation was 0.0554 wt%, with a coefficient of determination (R^2^) of 0.9103. These studies collectively confirm that optimizing data quality through appropriate preprocessing and feature engineering methods is essential for constructing robust and efficient LIBS machine learning models.

To the best of the authors’ knowledge, research on the classification of uranium slag samples using LIBS combined with machine learning algorithms remains relatively scarce. Therefore, to fill this research gap and evaluate the impact of feature engineering methods on classification performance within the LIBS-machine-learning framework, this study conducted a rapid classification analysis of nine types of uranium slag samples collected from three mining areas. The study systematically compares two feature engineering strategies (PCA and RF) based on datasets generated with no preprocessing and four spectral preprocessing methods—standard normal variate (SNV), Savitzky–Golay smoothing (SG), and their combined sequences SNV-SG and SG-SNV—under three different classification models (SVM, LDA, and KNN). All modeling processes were rigorously evaluated using five-fold cross-validation and parameter tuning was conducted through Bayesian optimization to ensure the robustness and reliability of the models. This study not only validates the feasibility of combining LIBS technology with machine learning for uranium slag classification but also provides a reliable technical solution for waste provenance tracing and risk management in nuclear environmental monitoring.

## 2. Materials and Methods

### 2.1. Sample Preparation

The U slag samples used in this study were collected from three mining areas in Jiangxi Province, with a total of nine groups. After the raw samples were crushed, ground, dried, and sieved through a 200-mesh screen, 5 g of each sample was weighed using a 0.0001 g electronic balance and then sealed for storage. Subsequently, the samples were pressed into circular discs with a diameter of 40 mm and a thickness of approximately 2.5 mm using a hydraulic press (pressing conditions: 8 MPa, 3 min), and numbered sequentially from 1# to 9#. In addition, the concentrations of U and Th in all samples were determined by ICP-MS in a single measurement, with the detailed results presented in Table 1.

### 2.2. Experimental Setup

**Figure 1:**
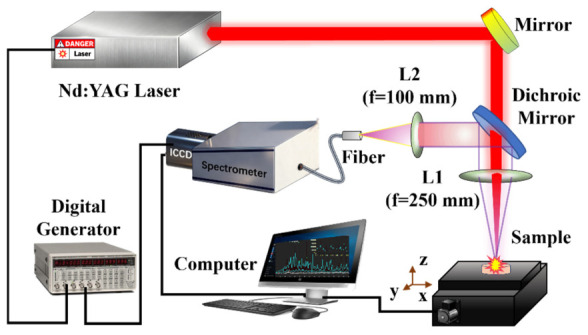
Schematic diagram of the experimental setup of LIBS.

The LIBS experimental setup employed in this study is illustrated in Figure 1, which mainly comprises a solid-state laser (TINY-D100L, GRACE LASER, Zhuolei Laser Technology Co., Beijing, China), a spectrometer equipped with an intensified charge-coupled device (ICCD) detector (Aryelle Butterfly, LTB, Edby Photonics Technology (China) Co., Beijing, China), a 3D sample translation stage, and a data acquisition and processing system. The laser has a central wavelength of 1064 nm, a pulse width of 6 ns, and a tunable pulse repetition rate within the range of 1–10 Hz. The maximum single pulse energy of the laser is 126 mJ (in this experiment, the laser energy and repetition frequency were set to 82.8 mJ and 10 Hz, respectively). The laser was focused onto the sample surface using a plano-convex quartz lens with a focal length of 250 mm. Under the operating conditions employed, the measured laser spot diameter on the sample surface was approximately 350 μm. The spectrometer features two channels, with spectral coverage ranges of 190–430 nm and 425–790 nm, and a resolution (λ/∆λ) of 50,000. In this experiment, the channel with a wavelength range of 190–430 nm was used. To obtain plasma spectral signals with a high signal-to-background ratio (SBR) and enhance the LIBS data quality, the Th II 387.12 nm line, which is less affected by interference and exhibits higher spectral intensity, was chosen as the reference line during the preliminary experiments. The variation of the SBR of this line was studied with respect to the ICCD detection delay time ($t_{d}$) and exposure gate width ($t_{w}$). The optimal values for $t_{d}$ and $t_{w}$ were determined to be 2.0 µs and 10 µs, respectively.

Under the optimal experimental parameter conditions, each group of samples was measured 30 times, with each measurement accumulating 50 pulses. A total of 270 LIBS spectra were obtained from the nine groups of samples, which were used for the subsequent modeling and performance evaluation.

### 2.3. Methods of Analysis

**Figure 2:**
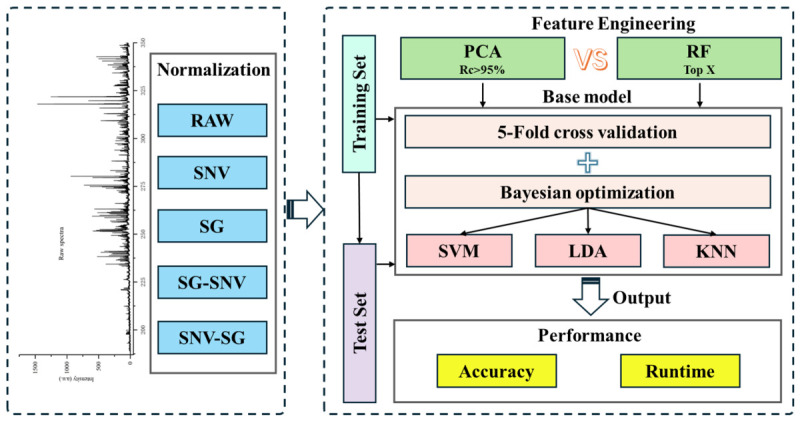
Flow chart for uranium slag classification based on LIBS combining with machine learning.

This study systematically evaluates the classification performance of LIBS combined with machine learning algorithms for uranium slag, focusing on comparing how different feature engineering methods coupled with classification algorithms affect prediction accuracy and computational efficiency. The overall analysis framework is shown in Figure 2, and the specific process is as follows:

1. Spectral Preprocessing: Based on the raw LIBS spectral data of nine slag samples, four normalization preprocessing methods were applied: SNV, SG, and their combinations SG-SNV and SNV-SG. The preprocessed data and raw data were stratified and split in a 7:3 ratio to construct the training and test sets, providing the data foundation for subsequent modeling.
2. Feature Engineering Methods: Feature extraction was performed using PCA and RF. In PCA, the PCs with a cumulative variance contribution rate exceeding 95% were selected as new features to achieve dimensionality reduction of the high-dimensional spectral data (spectral data points were reduced from 49,242 dimensions to at least 137 dimensions). Meanwhile, RF evaluated variable importance based on out-of-bag (OOB) error and selected the top X features (the number of X was determined to be 50 through subsequent analysis) as the input variables for the models, ensuring a significant reduction in spectral data dimensions while maintaining feature discriminability.
3. Hybrid Model Training and Optimization: PCA and RF were combined with three classification algorithms: SVM, LDA, and KNN, forming hybrid classification models. To improve model performance and prevent overfitting, five-fold cross-validation was used for initial evaluation, followed by Bayesian optimization to autonomously tune key hyperparameters and achieve the optimal model configuration.
4. Model Performance Evaluation: The model performance was evaluated from two dimensions: classification accuracy and computational efficiency. The classification accuracy of each model was compared on both the training and testing sets, and the overall runtime of the models was recorded to comprehensively evaluate their discriminative ability, generalization performance, and computational efficiency.

#### 2.3.1. Data Preprocessing

Appropriate preprocessing strategies can significantly enhance the training effectiveness and stability of the corresponding classifiers by ensuring that the acquired signals accurately reflect the chemical composition of the sample, whereas improper handling may distort class distribution and adversely affect classifier performance [32]. In this study, four preprocessing methods, including SNV, SG, and their combinations (SNV-SG and SG-SNV), were applied to the spectral data of uranium slag samples, and their performance was evaluated across various algorithm models.

SNV is a normalization method based on individual spectra. Its core principle is to centralize and scale the intensity values of each spectrum, transforming them into a standard normal distribution with a mean of 0 and a standard deviation of 1 [33]. This approach effectively eliminates systematic errors caused by baseline drift and light scattering, improving the signal-to-noise ratio and enhancing the discriminability of effective spectral features. The basic formula is:

(1) $${\hat{I}}_{i}=\frac{I_{i}-\mu }{\sigma }$$

where $I_{i}$ is the original spectral intensity, μ is the mean spectral intensity, and σ is the standard deviation of the spectral intensity.

SG is a signal processing algorithm based on local polynomial least squares fitting [34]. This method smooths the spectral data by performing polynomial fitting within a sliding window, effectively removing random noise while preserving key morphological features of the signal, such as peak shape and peak width. The specific formula is:

(2) $${\hat{I}}_{i}=\sum_{j=-k}^{k}c_{j}\cdot I_{i+j}$$

where $c_{j}$ is the SG filter coefficient, $I_{i+j}$ is the original intensity value of the *i+j*-th data point, and ${\hat{I}}_{i}$ is the spectral intensity value of the *i*-th data point after SG smoothing.

#### 2.3.2. Feature Engineering

Feature engineering is a key model regularization technique in machine learning, aimed at addressing the curse of dimensionality caused by high-dimensional data. By systematically selecting the most discriminative feature subset for the target variable and eliminating irrelevant and redundant features, overfitting is effectively suppressed, and model generalization and robustness are enhanced. In this study, each measurement collects a single spectrum with 49,242 spectral points, resulting in a matrix with approximately 1.3 × 10^7^ spectral points for a total of 270 spectra. Processing such a large matrix is not only cumbersome but also prone to causing system lag or even failure. Additionally, the abundance of redundant information and noise information in the spectra can adversely affect the classification results. Therefore, PCA and RF were used in this study for feature extraction and selection, applying both unsupervised and supervised evaluation methods to achieve dimensionality reduction and feature extraction for high-dimensional data.

PCA, as a classic unsupervised dimensionality reduction method, is primarily based on orthogonal transformation to map high-dimensional data into a lower-dimensional space, while retaining the key information of the data to the maximum extent [35]. In this study, the covariance structure of the spectral data was used to perform eigenvalue decomposition and extract the PCs that represent the direction of maximum variance. The number of effective components was determined based on the cumulative variance contribution rate, allowing for the removal of redundant information and noise interference, while preserving the main variance information of the data. This process provides a more concise data representation for subsequent classification modeling.

RF, as a supervised ensemble learning algorithm, focuses on quantifying the contribution of each feature to the model’s predictive performance [36]. In this study, during the model training process, a random forest classifier containing 200 decision trees was constructed by setting a fixed random seed. The feature importance was internally validated using OOB data. By randomly permuting a feature value and measuring the increase in prediction error, the contribution of that feature to classification accuracy was assessed. This method not only achieves dimensionality reduction of high-dimensional data but also enhances the model’s robustness by suppressing noise information. From a functional perspective, RF can be used not only for feature-importance evaluation but also as a standalone classifier for discrimination tasks. In this study, RF was directly applied to classification, and the corresponding results under different preprocessing strategies are summarized in Table 2. It can be observed that, although near-saturated training accuracy approaching 100% is achieved by RF, pronounced fluctuations are exhibited on the test set, with accuracies ranging from 85.19% to 96.30%. Moreover, the test performance was generally inferior to that obtained by subsequent hybrid frameworks in which RF-based feature selection was combined with other classifiers (SVM, LDA, and KNN). These results indicate that, when high-dimensional and complex LIBS spectral data are analyzed, the training data can be fitted effectively by RF as an independent classifier, whereas limited generalization is often encountered. Model performance is readily affected by spectral noise and redundant features, suggesting an elevated risk of overfitting. Therefore, RF was not treated as the primary classifier for in-depth discussion in this work. Instead, its advantages in feature-importance ranking and supervised dimensionality reduction were leveraged. RF was used as an efficient feature-selection tool to provide compact, highly discriminative inputs for downstream classifiers with stronger decision boundaries and better generalization, thereby enhancing the overall robustness and computational efficiency of the classification system.

#### 2.3.3. Classification Algorithms

In this study, to improve classification performance, three machine learning algorithms (SVM, LDA, and KNN) were combined with two feature engineering methods (PCA and RF) to construct classification models for uranium slag. Among them, SVM, as a supervised learning algorithm, works by mapping the original features into a high-dimensional feature space using a kernel function, and then constructing an optimal classification hyperplane within this space to maximize the margin between classes [37]. LDA, also a supervised classification method, constructs a discriminant function by maximizing the ratio of between-class variance to within-class variance, thereby achieving dimensionality reduction and classification [38]. KNN, as a lazy learning paradigm, classifies samples based on distance metrics by retrieving the K nearest samples in the training set and using majority voting or distance-weighted voting to determine the label of the unknown sample [39].

**Figure 3:**
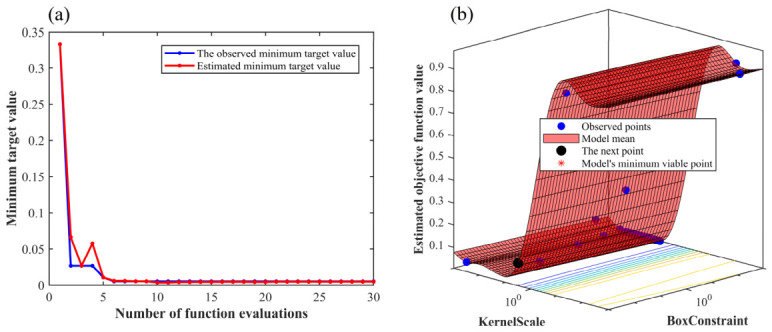
Bayesian optimization process. (**a**) Function calculation process; (**b**) Objective function model.

To improve model performance and generalization ability, all integrated models were initially evaluated using five-fold cross-validation, followed by further hyperparameter tuning through Bayesian optimization. The optimization process was set to a maximum of 30 evaluation iterations, aiming to minimize the classification error rate and determine the optimal configuration for each algorithm. All the parameters used for each algorithm are shown in Table 3. The Bayesian optimization procedure for the RF–SVM model is illustrated in Figure 3. As shown in Figure 3a, the optimization process rapidly converges toward the target value of 0.0059 by the sixth evaluation, after which the objective curve gradually plateaus. After all 30 function evaluations are completed, the result remains stable, indicating that the optimal region in the hyperparameter space can be efficiently identified by Bayesian optimization. Figure 3b further presents the surrogate modeling of the objective function over the hyperparameter space, where the distributions of observed samples, the predicted mean of the model, and the next evaluation point provide an intuitive visualization of the effective trade-off between global exploration and local exploitation. The optimal hyperparameters are ultimately determined as BoxConstraint = 997.7 and KernelScale = 23.72. With this configuration, the lowest classification error rate on the test set is obtained, thereby corroborating the effectiveness and reliability of Bayesian optimization for automated and precise hyperparameter tuning.

### 2.4. Model Evaluation

This study selects classification accuracy and computation time as the evaluation metrics for model performance. Classification accuracy reflects the proportion of correctly predicted samples to the total samples. The higher the value, the better the classification performance of the model. The specific calculation formula is as follows:

(3) $$\mathrm{Accuracy}=\frac{\mathrm{TP}+\mathrm{TN}}{\mathrm{TP}+\mathrm{TN}+\mathrm{FP}+\mathrm{FN}}$$

where TP denotes True Positives, TN denotes True Negatives, FP denotes False Positives, and FN denotes False Negatives.

## 3. Results and Discussion

### 3.1. Spectral Analysis

**Figure 4:**
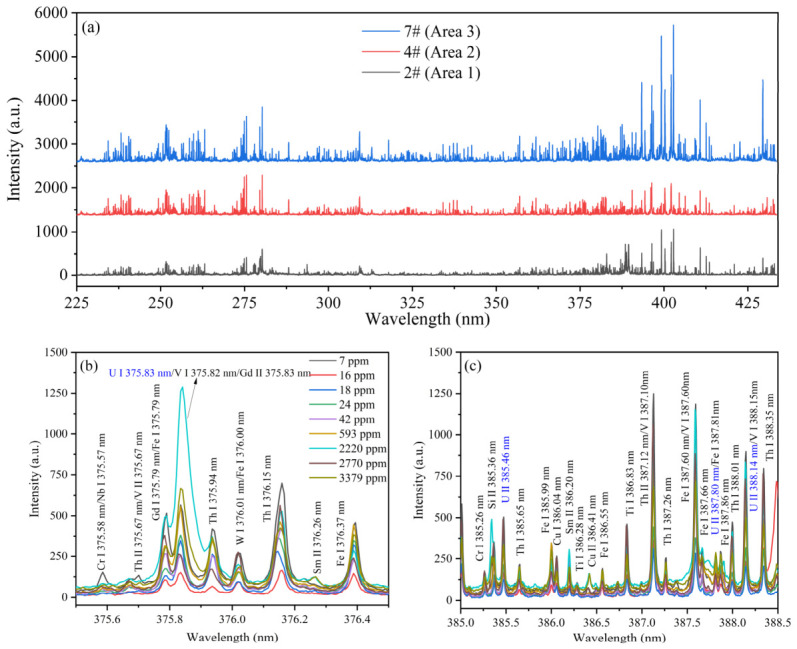
The raw LIBS spectra of uranium slag samples: (**a**) 187–434 nm; (**b**) 375.5–376.5 nm; (**c**) 385–385.5 nm.

As shown in Figure 4a, the raw average LIBS spectra of three representative uranium slag samples (2# from Area 1, 4# from Area 2, and 7# from Area 3) exhibit high complexity, with numerous emission lines densely distributed across the measured wavelength range of 225–434 nm. Notably, the spectral features of these three samples display pronounced differences among the three mining areas, reflecting distinct elemental compositions and relative abundances. Nevertheless, the dense packing of emission lines and the presence of spectral overlaps still render direct visual identification of category-specific lines challenging. Despite this complexity, several characteristic emission lines of uranium can be discerned. Specifically, emission peaks at 375.83 nm, 385.46 nm, 387.80 nm, and 388.14 nm are identifiable as characteristic U I and U II transitions. However, as highlighted in the magnified views of Figure 4b,c, these signals often represent composite intensities where uranium lines are intermingled with unresolved contributions from matrix elements such as Th, Fe, V, and Gd. This congestion, compounded by matrix effects and/or self-absorption, results in a pronounced nonlinearity between elemental concentration and spectral response. This behavior is further exacerbated by the dense spectral interference from thorium, which is highly excitable and frequently obscures the target uranium signals.

This intricate spectral landscape underscores the inherent difficulty of direct interpretation. The nonlinear response and multi-element interference preclude the effectiveness of simple intensity-based analysis, thereby necessitating the adoption of machine learning methods to achieve accurate origin tracing and robust category classification of uranium slag.

### 3.2. The Influence of the Characteristic Variables

#### 3.2.1. Unsupervised PCA Exploration

**Figure 5:**
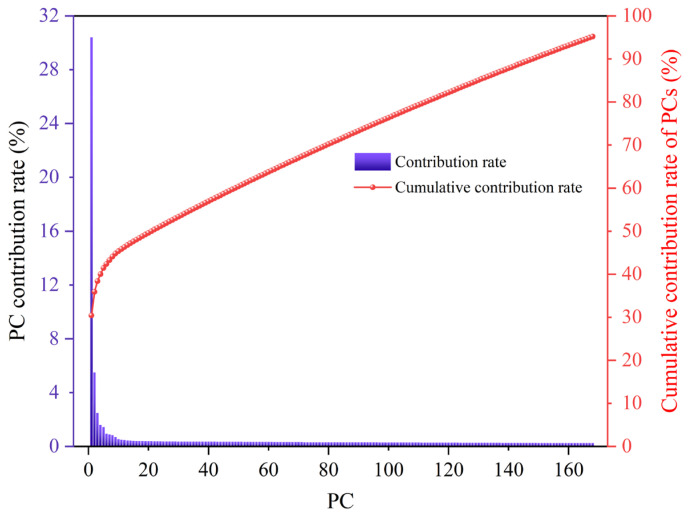
The variance contribution and cumulative contribution rates of the first 168 PCs.

**Figure 6:**
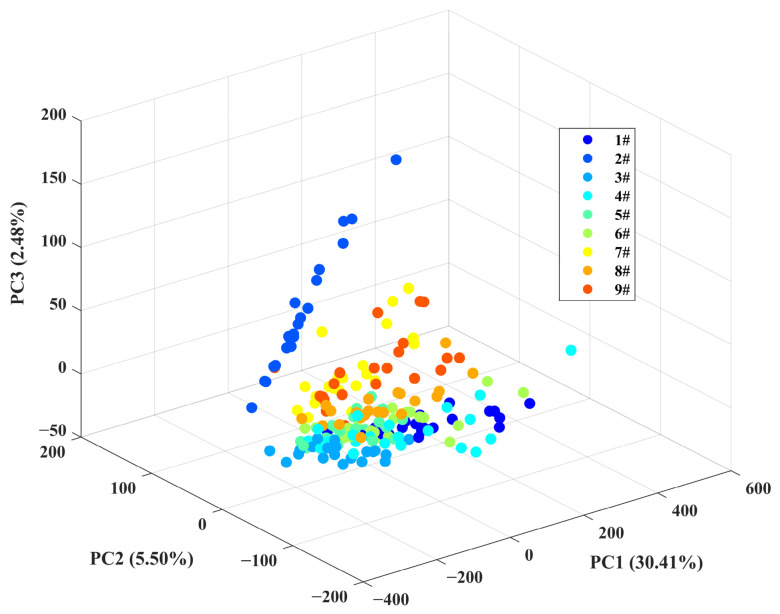
The 3D scatter plots of the first three PCs.

Figure 5 illustrates the variance contribution rates and cumulative contribution rates of the first 168 PCs after dimensionality reduction of the raw spectral dataset using PCA. It can be observed that the variance contribution rates of the first three PCs are 30.41%, 5.50%, and 2.48%, respectively. As the number of PCs increases, the variance contribution rate rapidly declines. When the number of PCs reaches 20, the contribution rate is only 0.39%, and the cumulative variance contribution rate of the first 20 PCs is just 49.60%. It is not until the number of PCs reaches 168 that the cumulative contribution rate reaches 95.20%. This indicates that the high-dimensional LIBS spectral data in this study has a complex structure, with information being relatively dispersed. It is clear that a small number of PCs by themselves cannot fully represent all spectral features, and a larger number of components is required to effectively capture the key information. Therefore, in the subsequent model training, at least the first 137 PCs selected by PCA (168, 204, 154, 138, and 137 for Raw, SNV, SG, SNV-SG, and SG-SNV) will be used as input features to ensure that 95.20% of the spectral variation information is included, providing a sufficient data foundation and discriminative basis for the classification model. Figure 6 further presents a three-dimensional scatter plot based on the first three PCs. It can be visually observed that the nine sample groups are widely dispersed in the space, with no clear clustering effect. Except for category 2#, which shows a relatively clear classification boundary with other categories, the overlap between samples of the remaining categories is quite significant, making effective classification difficult.

**Figure 7:**
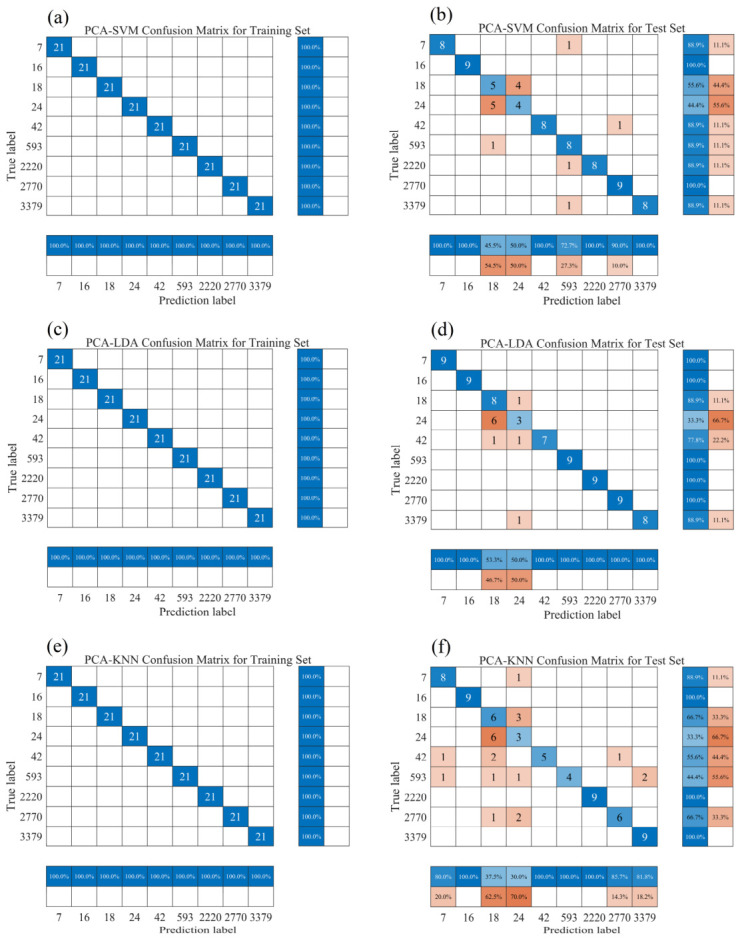
Classification results based on PCA feature extraction. (**a**,**b**) PCA-SVM; (**c**,**d**) PCA-LDA; (**e**,**f**) PCA-KNN.

Figure 7 shows the classification performance of three classification models that integrate PCA feature extraction on the raw spectral dataset. The classification accuracy of all three algorithms on the training set reached 100%. However, the classification performance on the test set was not ideal, indicating that features extracted solely based on PCA have limited discriminative power in the raw spectral data. Among the three algorithms, the LDA model performed the best but still misclassified a total of 10 samples from four categories, resulting in an overall accuracy of only 87.65%. The SVM model misclassified 14 samples, with an accuracy of 82.72%. The KNN algorithm produced the worst classification results. Except for the correct recognition and recall of categories 2#, 8#, and 9# (16, 2220, and 3379 ppm), 22 samples from the remaining six categories were misclassified, with notable misclassification occurring in category 5# (24 ppm) from the second mining area, where 6 samples were incorrectly identified as the category 4# (18 ppm) from the same area, leading to a recall rate of only 33.3% for category 5#. Additionally, 4 samples from categories 3#, 6#, and 7# (2770, 42, and 593 ppm) were misclassified as category 4#, resulting in a final precision of only 37.5% for category 4#. These results indicate that for raw spectral data without preprocessing, the discriminative ability of models based on PCA combined with various classification algorithms is limited, and all integrated models exhibit insufficient generalization capability. Therefore, appropriate spectral preprocessing and the integration of other feature engineering strategies need to be introduced to optimize the quality of input features, thereby further enhancing the discriminability and robustness of the classification models.

#### 3.2.2. Supervised RF Exploration

**Figure 8:**
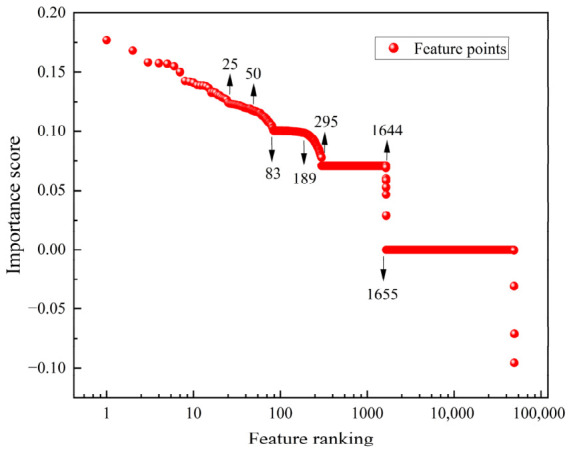
The distribution map of the importance scores of spectral points.

Figure 8 shows the importance scores of the 49,242 spectral points ranked in descending order using the RF method. It can be observed that the distribution of importance scores exhibits a typical “long tail” characteristic, where only a few feature points exhibit significant discriminative power. Among them, the feature point ranked top 1 has an importance score (IS) of only 0.177, which then gradually decreases. By the time the rank reaches 83, the IS drops below 0.10. At ranks 83 and 189, a significant IS decline occurs, and after rank 295, the IS values of the feature points remain at a relatively low and stable level. Notably, the feature point ranked 1644 becomes the key node where the IS transitions from positive to zero or even negative values. This distribution pattern indicates that, among the 49,242 spectral points, only the top 1600 or so feature points contribute positively to the model, with most of the effective information concentrated in the top 200 feature points. The subsequent feature points have a limited effect on improving the model performance and may even exert a negative influence. Therefore, by selecting key features ranked higher, the data dimensionality can be reduced by more than two orders of magnitude while retaining the majority of the effective information, thus significantly improving the computational efficiency of the model.

**Figure 9:**
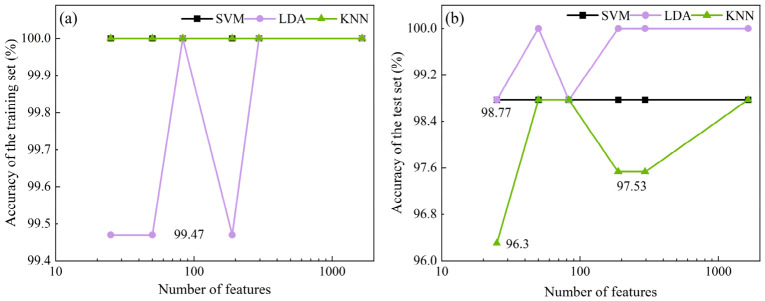
Classification results based on the selection of variables using RF method. (**a**) Training set; (**b**) Test set.

Based on the RF feature importance evaluation results, features ranked in the top 25, 50, 83, 189, 295, and 1644 were selected as input variables and analyzed using three classification models: SVM, LDA, and KNN. The specific classification results are summarized in Figure 9. On the training set, all models perform excellently, with both the RF-based SVM and KNN hybrid models achieving an accuracy of 100%. Although LDA does not reach a perfect 100% accuracy across all feature dimensions, its accuracy remains above 99.47% even under low-dimensional conditions (with the top 25 or 50 features), demonstrating strong feature utilization efficiency and stable classification performance. However, on the test set, the models exhibit different generalization characteristics. SVM is not sensitive to changes in input dimensionality, maintaining a stable accuracy of 98.77% without fluctuation as the number of features increased. The LDA model achieves 100% accuracy on the test set when the feature count reaches 50, 189, 295, and 1644, showing a trend of improved performance as the feature dimension increases. In contrast, when the number of features increases from 25 to 50, the accuracy of the KNN model significantly improves from 96.3% to 98.77%. However, when the feature count exceeds 83, the test accuracy shows slight fluctuations, even experiencing a minor decline, indicating a potential overfitting risk. It is noteworthy that when all 1644 features are used, the test performance of all classification algorithms does not significantly outperform the lower-dimensional configurations, further emphasizing the critical impact of controlling input dimensionality on model generalization ability. Considering the performance of each algorithm on both the training and test sets, as well as potential computational efficiency, the top 50 features are selected as input variables for the classification models, achieving an optimal balance between classification accuracy and feature representativeness.

### 3.3. Comparison of Classification Accuracy

**Figure 10:**
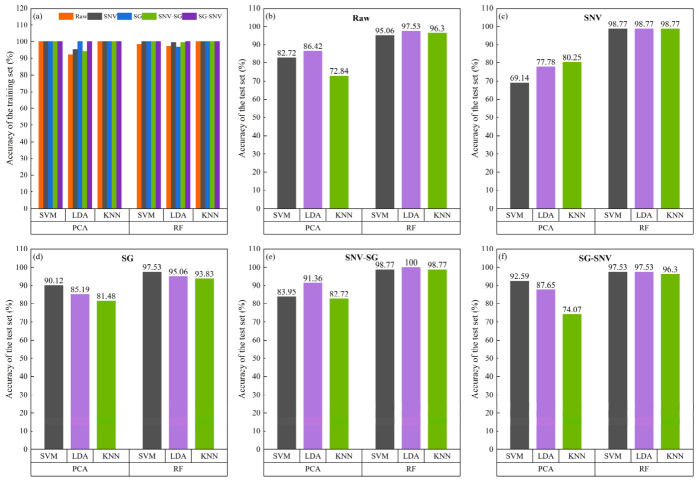
The classification accuracy of the hybrid model on the training set and the test set. (**a**) Hybrid models; (**b**) Raw; (**c**) SNV; (**d**) SG; (**e**) SNV-SG; (**f**) SG-SNV.

Figure 10 shows the classification accuracy of various hybrid models based on two feature engineering methods, evaluated on both the training and test sets. From the training set results shown in Figure 10a, it can be seen that the vast majority of the hybrid models demonstrate very high discriminative performance, with accuracies all exceeding 98.77%, indicating that the models are able to fully learn the discriminative information from the spectral features and exhibit good fitting during training. However, some models still show significantly lower performance. Using the raw data, the LDA model based on PCA feature extraction achieves a training accuracy of only 92.06%, which is clearly inferior to the other combinations under the same condition. This suggests that PCA may have lost some key spectral information critical to the classification task during the feature compression process, preventing the model from fully capturing the intrinsic structure of the data. In contrast, the corresponding models based on RF feature selection consistently maintain higher accuracies on the training set.

Figure 10b–f correspond to the prediction results for the raw data and the four preprocessed datasets. It can be clearly observed that, regardless of the preprocessing method, models integrated with RF feature selection performed markedly better than those integrated with PCA. Specifically, the accuracies of models under RF feature selection are all higher than 95.06%, whereas PCA-based models fall below this value in most cases, with only a few instances exceeding 90%. In addition, preprocessing is found to noticeably improve the classification accuracy of most hybrid algorithms compared with the use of raw data. Under the SNV-SG preprocessing condition in particular, the RF-based classification models are outstanding, with all algorithms exceeding 98.77% accuracy and the LDA hybrid model achieving 100%, demonstrating very strong discriminative performance. Meanwhile, the classification results obtained with different preprocessing combinations (SNV-SG and SG-SNV) show slight differences, indicating that the performance of the models is influenced by the order of spectral preprocessing methods, which has also been reported in the referenced study [40]. It is noteworthy that the PCA method selects at least 137 PCs as feature variables (168, 204, 154, 138, and 137 for Raw, SNV, SG, SNV-SG, and SG-SNV, respectively), whereas RF obtains superior classification with only 50 feature variables. This result indicates that RF is more efficient at suppressing redundant features while retaining key spectral information. It also reflects the structural advantages of RF in mitigating the curse of dimensionality and enhancing model generalization.

### 3.4. Comparative Analysis of Feature Engineering Mechanisms

A notable distinction between the two feature engineering strategies lies in the dimensionality required to achieve optimal classification performance. RF-based selection consistently attained superior accuracy using only the top 50 ranked features, whereas PCA required at least 137 PCs to reach a cumulative variance contribution exceeding 95%, irrespective of preprocessing. This disparity reflects fundamentally distinct feature extraction mechanisms.

**Figure 11:**
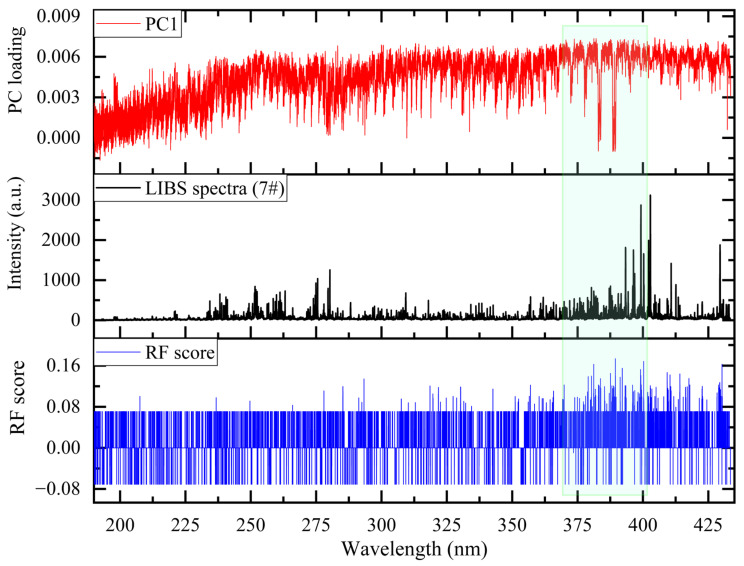
The distribution of PCA loadings and RF importance scores across spectral data points.

Figure 11 provides a direct comparison between RF-identified features and PCA-extracted components. The upper panel displays the PC1 loading, the middle panel shows a representative LIBS spectrum (7#), and the lower panel presents RF importance scores. The green box indicates the wavelength range where the top 50 RF-selected features are predominantly concentrated, specifically within the 370–400 nm range, aligning precisely with characteristic emission lines. A striking contrast is evident: the PC1 loading exhibits a broad, slowly varying structure, whereas RF importance scores are concentrated at discrete spectral positions coinciding with element-specific emission lines. By assigning high importance to these spectrally meaningful features, RF effectively isolates discriminative information while suppressing noise.

This contrast directly accounts for the observed performance gap. RF leverages class-label information to concentrate discriminative power into a compact set of interpretable spectral features—characteristic emission lines reflecting elemental composition differences. PCA, by contrast, operates without class labels, extracting components that maximize overall spectral variance. The high dimensionality and densely packed spectral features of LIBS data further amplify this divergence. Within a high-dimensional sparse structure, unsupervised PCA cannot distinguish classification-relevant spectral lines from non-discriminatory but high-variance interferences, forcing discriminative information to be dispersed across numerous components. RF, guided by class labels, possesses a natural capacity for sparse selection, accurately pinpointing critical spectral lines from the dense “forest” of features.

Thus, RF-based feature selection achieves superior performance with far fewer features by identifying interpretable, class-discriminative spectral features, whereas PCA requires a substantially larger set that includes variance irrelevant to the classification task. This advantage, combined with computational benefits, underscores the suitability of RF-based feature selection for robust LIBS-based classification of complex nuclear materials such as uranium slag.

As summarized in Table 4, the top 50 features selected by the RF-based method are predominantly concentrated within the 370–400 nm range, aligning with the green box in Figure 11 and indicating that the most discriminative spectral information is localized within this narrow region.

Due to the RF algorithm evaluating importance on a per-spectral-point basis, adjacent points within the same emission peak may yield varying importance scores, resulting in repeated assignments to identical spectral lines. Accordingly, these 50 features correspond to 37 unique spectral lines. From the perspective of elemental composition, the identified key characteristic lines primarily originate from radioactive elements such as U and Th, as well as Fe, Ca, and various rare earth elements (e.g., Gd, Dy, Er, Nd). These spectral lines do more than just reflect the complex chemical makeup of the uranium slag samples; they serve as the core discriminative criteria for classification—constituting the characteristic “spectral fingerprints” of uranium slag from different origins. The concentration of high-importance features on these specific emission lines demonstrates that the RF algorithm can precisely capture class-distinguishing spectral variations. This effectively enhances both the model’s interpretability and its classification performance.

### 3.5. Analysis of Computational Efficiency

**Figure 12:**
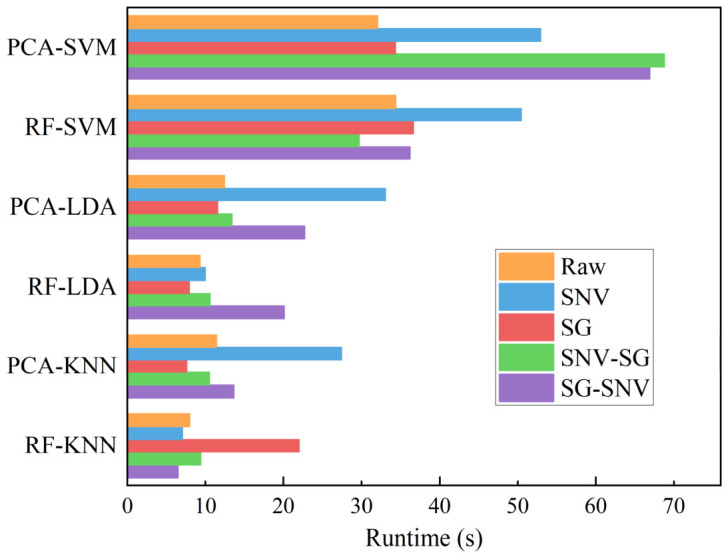
The runtime of each hybrid model.

Figure 12 presents the runtime comparison across the 30 hybrid models. As can be seen, SVM-based models consistently require the longest processing times, reaching up to 67 s, regardless of the feature engineering method employed. This is because SVM constructs the optimal separating hyperplane by solving a quadratic programming problem, a process that becomes increasingly computationally demanding as both the number of samples and the feature dimensionality increase. Consequently, SVM is inherently more time-consuming than the other classifiers evaluated. In contrast, LDA operates via eigenvalue decomposition, which is computationally lighter and scales more favorably with feature dimensions. KNN, as a lazy learner, incurs negligible training cost and performs prediction solely through distance calculations, resulting in substantially lower computational overhead. These algorithmic characteristics are empirically reflected in the results: LDA and KNN models completed within 13 s in most cases.

The choice of feature engineering method also has a substantial influence on runtime. RF-based selection retained only 50 key features per spectrum, whereas PCA required at least 137 PCs to achieve a cumulative variance contribution exceeding 95%. This disparity in input dimensionality directly affects computational cost, particularly for classifiers whose complexity is sensitive to the number of features. For SVM, reducing the feature count from over 137 to 50 lowered the runtime from above 40 s (PCA-SVM) to approximately 27–35 s (RF-SVM). For KNN, the effect was even more pronounced: RF-KNN achieved sub-10 s runtimes under all preprocessing conditions except SG, whereas PCA-KNN consistently exhibited longer processing times. These observations confirm that effective dimensionality reduction is a key factor in enhancing computational efficiency.

Preprocessing introduces a secondary but non-negligible influence on runtime. Models incorporating SG-based preprocessing generally required slightly longer execution times compared to those using raw or SNV alone. This increase can be attributed to the computational overhead of the SG smoothing operation, which performs polynomial fitting within a sliding window across each spectrum. Nevertheless, the magnitude of this effect is minor relative to the differences driven by classifier choice and feature engineering, indicating that preprocessing methods can be selected primarily on the basis of classification performance without incurring prohibitive computational costs.

Collectively, these results demonstrate that runtime differences among hybrid models primarily originate from the intrinsic complexity of the classifier and the dimensionality of the input feature set, with preprocessing playing a secondary role. Notably, the RF-LDA model with SNV-SG preprocessing achieved 100% accuracy with a runtime of only 10.46 s, illustrating that careful selection of both feature reduction and classifier is crucial for constructing efficient, high-performance LIBS-based classification systems suitable for large-scale or real-time applications.

## 4. Conclusions

In this study, the feasibility and effectiveness of combining LIBS with multiple machine learning algorithms for origin identification and category classification of nine uranium slag groups from three mining areas were systematically evaluated. The influence of different feature engineering methods and spectral preprocessing strategies on classification performance was analyzed in detail. Based on the raw spectra and the spectra preprocessed by SNV, SG, and the combined sequences SNV-SG and SG-SNV, three algorithms, namely SVM, LDA, and KNN, were integrated with two feature extraction methods, PCA and RF, to construct hybrid classification models. The results indicate that feature engineering plays a critical role in enhancing discriminative ability and optimizing computational efficiency. Compared with PCA, RF-based hybrid models more effectively identified and retained spectrally informative features that are most relevant to the classification task, while markedly suppressing redundancy and noise. With only the top 50 features retained, RF achieved superior classification across multiple preprocessing conditions, whereas PCA required at least 137 PCs, demonstrating the advantage of RF in feature compression and information retention. Under the SNV-SG condition in particular, the RF-LDA model achieved 100% accuracy on the test set with a runtime of only 10.46 s, indicating excellent discriminative performance and high computational efficiency.

In summary, the study confirms that the combination of LIBS and machine learning possesses substantial application potential for rapid classification and category identification of uranium slag. The central role of feature engineering in improving model performance, enhancing interpretability, and increasing runtime efficiency was highlighted. The study provides a reliable methodological basis for the identification and risk control of radioactive waste in nuclear environmental monitoring, and this approach holds practical significance for advancing the secure utilization of nuclear resources and the management of radiological environments.

## Figures and Tables

**Table 1:** U slag category numbers and the corresponding concentrations of U and Th elements.

Concentration (ppm)	Category No.								
	Area 1			Area 2			Area 3		
	1#	2#	3#	4#	5#	6#	7#	8#	9#
U	7	16	2770	18	24	42	593	2220	3379
Th	555	429	12	165	88	78	34	41	335

**Table 2:** Classification results of the RF model.

Preprocessing	Training Set	Test Set	Running
	Accuracy (%)	Accuracy (%)	Time (s)
Raw	100	85.19	22.6459
SNV	100	96.30	15.3051
SG	99.47	93.83	29.2269
SNV-SG	100	95.06	29.835
SG-SNV	100	93.83	32.7128

**Table 3:** The main parameters of the classification models.

Model	Main Parameter
SVM	BoxConstraint = 997.7; KernelScale = 23.72
LDA	DiscrimType = diagLinear; Delta = 1.0481 × 10^−6^; Gamma = 0.019666
KNN	NumNeighbors = 10; Distance = cityblock; DistanceWeight = inverse

**Table 4:** The characteristic spectral lines corresponding to the top 50 features of RF feature selection. (“/” denotes overlapping peaks or co-existing spectral lines that could not be fully resolved).

Feature Ranking	Spectral Line	Feature Ranking	Spectral Line	Feature Ranking	Spectral Line
1	Gd II 389.47	18	U II 399.25/V I 399.26/ Gd I 399.26	35	Ca II 393.36
2	V II 400.29/Fe II 400.29	19	Mg I 383.82	36	Ca II 393.36
3	U I 381.19/Th II 381.19	20	Mn II 293.30/Th II 293.30	37	Th I 402.86/Fe II 402.86
4	Ca I 430.25	21	Er I 409.29	38	U II 399.25/V I 399.26/ Gd I 399.26
5	Th II 392.03/Fe I 392.02	22	U II 399.25/V I 399.26/ Gd I 399.26	39	U II 388.14
6	Sm I 399.00	23	U II 399.25/V I 399.26/ Gd I 399.26	40	V II 383.23/Th I 383.23/ Cr I 383.23
7	Th II 392.03/Fe I 392.02	24	Fe I 429.41	41	Nb I 388.60
8	Er I 409.29	25	Fe I 429.41	42	Eu I 318.55
9	Th II 387.12	26	Ce II 428.94	43	Nb I 388.60
10	Dy II 414.15	27	U II 399.25/V I 399.26/ Gd I 399.26	44	Fe I 429.41
11	Th II 410.36	28	Th II 382.94	45	Th II 410.36
12	U II 399.25/V I 399.26/ Gd I 399.26	29	Nd II 417.95	46	Th II 422.67
13	Gd II 389.47	30	Dy II 414.15	47	Th II 388.86
14	Th II 380.30	31	Fe I 369.81	48	Dy II 414.15
15	Fe I 379.06	32	Cu II 417.62	49	Cr II 285.22/Cu 285.21
16	Th I 391.30	33	Th II 396.13	50	Nd II 410.65
17	Cu II 417.62	34	Fe I 357.02/U I 357.02		

## Data Availability

Data will be made available upon reasonable request from the author.

## Abbreviations

The following abbreviations are used in this manuscript:

LIBS	Laser-induced breakdown spectroscopy
SNV	Standard normal variate
SG	Savitzky–Golay smoothing
PCA	Principal component analysis
RF	Random forest
SVM	Support vector machine
LDA	Linear discriminant analysis
KNN	K-nearest neighbors
AAS	Atomic absorption spectroscopy
ICP-OES/MS	Inductively coupled plasma optical emission spectroscopy
AUC	Area under the curve
PLSR	Partial least squares regression
DA	Discriminant analysis
GA	Genetic algorithms
CARS	Competitive adaptive reweighted sampling
SPA	Successive projections algorithm
LAR	Least angle regression
LR	Logistic regression
VIM-RF	Variable importance-based random forest
PLS	Partial least squares
LS-SVM	Least squares support vector machine
ICCD	Intensified charge-coupled device
SBR	Signal-to-background ratio
OOB	Out-of-bag error
PCs	Principal components
IS	Importance-score
